# Clinical epidemiology of snakebite envenoming in hospitals 11 provinces of Yangtze River Basin and southern regions of China: A retrospective hospital-based analysis

**DOI:** 10.1371/journal.pntd.0013247

**Published:** 2026-04-27

**Authors:** Shijiao Yan, Wenning Fu, Zhangren Yan, Yanlan Hu, Xingyue Song, Juntao Wang, Wenjie Hao, Lanfen He, Yu Chen, Mohamed Diané, Ibrahima Sory Souaré, Wei Guo, Wenkai Bin, Yu Ma, Xiaotong Han, Chuanzhu Lv

**Affiliations:** 1 School of Public Health, Hainan Medical University, Haikou, Hainan, China; 2 Key Laboratory of Emergency and Trauma of Ministry of Education, Hainan Medical University, Haikou, Hainan, China; 3 School of Nursing, Tongji Medical College, Huazhong University of Science and Technology, Wuhan, Hubei, China; 4 Department of surgery of Traditional Chinese Medicine, Affiliated Hospital of Jiangxi University of Traditional Chinese Medicine, Nanchang, Jiangxi, China; 5 Department of Emergency, Hainan Clinical Research Center for Acute and Critical Diseases, The Second Affiliated Hospital of Hainan Medical University, Haikou, Hainan, China; 6 China-Guinea Friendship Hospital, Kipe Ratoma 030 BP710 Guinea Friendship Hospital, Conakry, Guinea; 7 Emergency Department, Beijing Tiantan Hospital, Capital Medical University, Beijing, China; 8 The Affiliated Nanhua Hospital, Department of Emergency, Hengyang Medical School, University of South China, Hengyang, Hunan, China; 9 Emergency Department, Chongqing University Central Hospital, Chongqing Emergency Medical Center, Chongqing, China; 10 Department of Emergency Medicine, Hunan Provincial Key Laboratory of Emergency and Critical Care Metabolomics, Hunan Provincial Institute of Emergency Medicine, Hunan Provincial People’s Hospital/The First Affiliated Hospital, Hunan Normal University, Changsha, Hunan, China; 11 Emergency Medicine Center, Sichuan Provincial People’s Hospital, University of Electronic Science and Technology of China, Chengdu, Sichuan, China; University of Liverpool, UNITED KINGDOM OF GREAT BRITAIN AND NORTHERN IRELAND

## Abstract

**Background:**

As the largest developing country, China works towards reducing mortality and disability from snakebite envenoming (SBE) by 50% before 2030, as epidemiological evidences are essential.

**Methods:**

A cross-sectional survey was conducted in 11 provinces in China using a multistage stratified cluster random sampling, and hospitalized snakebite victims with complete case histories from January 1, 2017, to December 31, 2021, were included.

**Results:**

A total of 40,817 snakebite victims were enrolled in this study. The most common snake species were Agkistrodon halys (16.04%) and Trimeresurus stejnegeri (11.40%). However, 56.36% of the snake species remained unidentified. Furthermore, our results revealed that 60.36% of snakebite victims were males, 63.71% were older than 50 years, 59.70% were peasants, 11.14% experienced moderate envenomation on admission, and 0.64% had severe envenomation on admission. Ultimately, only 6.82% of the victims achieved complete healing, whereas 82.44% of snakebite victims demonstrated improvement in their condition upon discharge. Only 69.57% of snakebite victims received antivenom treatments. Furthermore, age, occupation, hospitalization duration, snake specie, site of bite, location, activity, envenomation on admission, clinical manifestation, and treatment significantly affected the discharge outcomes of snakebite victims.

**Conclusions:**

This study is the first large-sample investigation focusing on the epidemiological characteristics of SBE in China, addressing research gaps and filling the void in the relevant the World Health Organization data. The findings call for authorities and managers to strengthen partnerships, coordination, and resources, reduce SBE incidents, and ensure that victims receive fair, safe, and effective treatments.

## Author summary

Our study provides authoritative and comprehensive epidemiological data of snakebite envenoming (SBE) and independent factors influencing discharge outcomes of snakebite victims, based on a multistage stratified cluster random sampling survey, including 11 provinces in China. A total of 40,817 snakebite victims from January 1, 2017, to December 31, 2021 were included in this study. The most common snake species were Agkistrodon halys and Trimeresurus stejnegeri. Furthermore, 60.36% of snakebite victims were males, 63.71% were older than 50 years, 59.70% were peasants, and 56.28% were bitten on feet, ankles, and calves. Only 32.45% of snakebite victims sought hospitals care within 2 hours of the incident and 69.57% received antivenom treatment. Furthermore, we explored the independent factors affecting the discharge outcomes of snakebite victims. Age, occupation, hospitalization duration, snake specie, site of bite, location, activity, envenomation on admission, clinical manifestation, and treatment significantly affected the discharge outcomes of snakebite victims. Our study is of important reference value for the subsequent in-depth research related to snakebite envenoming in China.

## Introduction

Presently, approximately 5.4 million people are bitten by snakes each year, resulting in 1.8-2.7 million cases of envenoming. This leads to 81,000–138,000 deaths and 400,000 survivors with permanent physical and psychological disabilities, making snakebite envenoming a substantial global public health challenge [1]. In 2017, the World Health Organization (WHO) added snakebite envenoming to its list of neglected tropical diseases (NTD); 2 years later, a strategy for the prevention and control of snakebite envenoming was launched to prevent the global toll of death and disability by 2030 [2,3].

Previous research showed that women, children, and farmers in impoverished rural areas of low-income and middle-income countries are most affected by snakebite envenoming, and that the burden of snakebite envenoming is heaviest in countries with the weakest health systems and the most unequal distribution of medical resources [4]. As the world’s largest developing country, China’s economy and healthcare system have developed rapidly over the past few decades [5]. However, with a large population base, a significant wealth disparity, and the influence of traditional attitudes, the high incidence of snakebite envenoming and the lack of standardized follow-up treatments remain serious problems [6]. In addition, China is a vast country with a diverse topography and a wide variety of climates, where extensive areas of vegetation cover provide good habitat conditions for snakes [7].

According to statistics, China hosts over 300 species of snakes and approximately 100 species of venomous ones [8]. The species richness shows a multi-peak pattern on both latitudinal and longitudinal gradients, with the highest richness in areas with subtropical and tropical monsoon climates in eastern and southern China [9]. According to statistics from the China Disaster Emergency Rescue Medical Association (2020), over 300,000 people are bitten by snakes annually [10]. However, data on snakebite envenoming are usually underestimated, primarily due to inadequate epidemiological reporting caused by the prevalence of snakebite envenoming in poor rural areas as well as insufficient research [6,11]. In addition, the National White Paper on Snakebite Epidemiology revealed that 55.47% of people with snakebite in China had residual sequelae, and 35.18% of them experienced disabilities including amputations [6]. As a preventable and curable disease, understanding its epidemiological distribution, proper prevention, and standardized treatments can significantly reduce the morbidity, disability, and mortality associated with snakebite envenoming.

However, epidemiological studies on snakebite envenoming in China are limited. Besides, the sample size of existing research is relatively small. One study in Taiwan, China, using a health insurance database from 2002-2014, revealed that 12,542 snakebite victims received antivenom therapy, averaging 965 cases annually and a crude incidence rate of 4.2 cases per 100,000 people [12]. An epidemiological study in the Wuling Mountains area of Chongqing, western China, involved 440 snakebite victims, with most victims being male [13]. Similarly, studies on snakebite envenoming in children in Hong Kong and envenomation by Naja atra found a higher proportion of male snakebite victims; besides, their sample sizes were small [14,15]. Previous research [16] indicated that the scarcity of data regarding the epidemiology, socioeconomic impacts, and disability burdens associated with snakebite envenoming results in limited evidence to guide treatment protocols. Consequently, there is an inability to allocate health resources appropriately based on the occurrence of snakebite envenoming. This is the primary reason for the current poor treatment and high disability rates of snakebite envenoming. Therefore, conducting a multicenter, large-sample epidemiological survey of snakebite envenoming in China is essential.

According to previous studies, factors associated with snakebite envenoming complications or death include incorrect perceptions of access to medical care, delayed access to medical care, poor prehospitalization care, incorrect identification of snake species, inadequate administration of antivenom, and lack of designated treatment guidelines, which are largely amenable to improvement or avoidance [17–20]. Among them, antivenom represents the sole effective treatment for preventing or reversing the toxic effects of snakebite envenoming, and the majority of deaths and severe consequences resulting from snakebite envenoming could be entirely avoided through broader provision of antivenom [21]. However, the current severe lack of epidemiological data on snakebite envenoming and venomous snakes species hinders the estimation of specific demand. Additionally, the absence of appropriate distribution policies results in regional disparities in the availability of antivenom [6].

Given the inadequacy of previous studies, the incidence and severity of snakebite envenoming in China have been underestimated and received little attention. Therefore, in this research, a multicenter, large-sample epidemiological survey, including 11 provinces in China, was conducted to explore the an epidemiological characteristics of snakebite envenoming and to analyze the independent factors influencing hospital discharge outcomes among snakebite victims. Our research findings are expected to provide a reference for the future policies, interventions, and standard treatments of snakebite envenoming, thereby contributing to accomplish the strategic goal of halving the disability and mortality rates of snakebite envenoming by 2030 in China.

## Methods

### Ethics statement

All the methods used in this study adhered to the principles of the Declaration of Helsinki. This study received approval from the Medical Ethics Committee of the Hainan Medical University (HYLL-2022–226), Haikou, China. Given the retrospective nature of this study and the absence of individual snakebite victim identities in the data collected, the victim’s consent was waived.

### Study area

The snakes are usually distributed within forests, grasslands, farmland, and other lands in tropical or subtropical zones of south China that have a warm and humid climate [22]. Therefore, based on comprehensive literature research and pragmatic considerations, 11 provinces/municipalities/autonomous regions in the Yangtze River Basin and southern regions of China, with subtropical or tropical monsoon climate, were included in this research to collect epidemiological data on snakebite victims, including the Hubei, Hunan, Guangdong, Fujian, Jiangxi, Sichuan, Guizhou, Hainan, and Yunnan provinces, Chongqing municipality, and Guangxi Zhuang Autonomous Region. In addition, these provinces possess a rich vegetation variety providing excellent snake habitats and have dense populations, high population density, and relatively underdeveloped economies primarily relying on agriculture. For example, Jiangxi Province is characterized by Jiangnan hills and mountains, with a convergence of water sources and a dense network of over 2,400 rivers [23]; Hunan Province is situated in the transitional zone from the Yungui Plateau to the Jiangnan Hills and Nanling Mountains, surrounded by mountains to the east, south, and west, featuring undulating hills and ridges in the central part, through which rivers such as the Xiangjiang and Yuanjiang flow [24,25]; Guizhou Province, located on the Yungui Plateau, comprises over 90% mountainous and hilly terrain, with numerous rivers from the Yangtze and Pearl River Basins [26].

### Design and setting

A national cross-sectional study was conducted in China from June 2022 to March 2023, employing a multistage stratified cluster random sampling to select the research sample. First, based on comprehensive literature research and practical considerations, 11 provinces/municipalities/autonomous regions with high incidence of severe snakebite incidents in the Yangtze River Basin and southern regions were selected: Central China (Hubei, Hunan); South China (Guangdong, Guangxi, Hainan); East China (Fujian, Jiangxi); and Western regions (Sichuan, Guizhou, Yunnan, Chongqing). Second, cities were stratified by economic development status (high, medium, low), with three prefecture-level cities (including one provincial capital) selected from each sampled province as the second-stage sampling units. Third, three counties were selected from each sampled prefecture-level city, also stratified by economic development status (high, medium, low), as the third-stage sampling units. In conclusion, based on the capacity to provide snakebite treatment services, a total of 116 hospitals were included in this study to collect data on snakebite victims (Table A in S1 File). The provincial tertiary Grade A hospitals admitted approximately one-fifth of the snakebite victims, primarily those with more severe conditions. In contrast, municipal tertiary Grade A hospitals and county-level tertiary Grade B hospitals treated a larger proportion of snakebite cases, with Traditional Chinese Medicine hospitals demonstrating particularly high victim volumes. This is because not all hospitals admit snakebite victims. Previously, snakebite victims were mainly treated in Traditional Chinese Medicine hospitals. The choice of hospital for medical treatment among the Chinese population is related to the perceived diagnostic and therapeutic specialties of the hospital as communicated through word-of-mouth among the local community. In addition, due to the fact that not every hospital maintained an adequate supply of antivenom, snakebite victims were relatively concentrated in certain hospitals. Moreover, in China, there is no specialized department for snakebite treatment in hospitals, and snakebite victims are admitted to various departments. Therefore, when collecting medical records, all inpatients with snakebite diagnoses were included from the hospital information system of each participating hospital.

The inclusion criteria for this study were hospitalized snakebite victims of all ages recorded in the hospital healthcare system with clinically diagnosed snakebite envenoming from January 1, 2017, to December 31, 2021, in the sampling units. Patients with incomplete medical records of snakebite envenoming were excluded. The following data were extracted: age, sex, occupation, marital status, province, discharge outcome, hospitalization duration, snake specie, site of bite, location, activity, envenomation on admission, clinical manifestation, medical treatment, and prehospital delay time (Table B in S1 File). The diagnosis of snakebite relies on a comprehensive assessment that encompasses the patient’s medical history, physical examination, relevant laboratory or imaging findings, and the epidemiological data specific to the geographical region where the snakebite occurred. Identifying the snake species responsible for the bite involves examining the characteristics of the snakebite, utilizing eyewitness accounts detailing the snake’s features, leveraging on-site photographs of the snake (if accessible), or inspecting physical evidence such as the actual snake involved or referring to images of common venomous snakes in the local vicinity for the victim to identify. Key attributes for identification include the snake’s size, length, weight, coloration, and body pattern characteristics [27]. Detailed definitions of other major variables in this study are provided in S1 File.

### Statistical analysis

Statistical analyses were performed using STATA/MP 17.0. Continuous variables are described as mean ± standard deviation (Mean ± *SD*), and categorical variables are described as frequencies (*n*) and percentages (%). Discharge outcomes were categorized into five groups: healing, improvement, unhealing, withdrawal, and death. For univariate analysis between groups, the Kruskal-Wallis test was used for continuous variables, and *χ*^2^ test was used for categorical variables. Factors significantly associated with disease outcomes were included in the multivariate logistic regression analysis to determine the independent risk factors for disease outcomes. Statistical significance was set at *P* < 0.05 (two-tailed).

## Results

**Fig 1:**
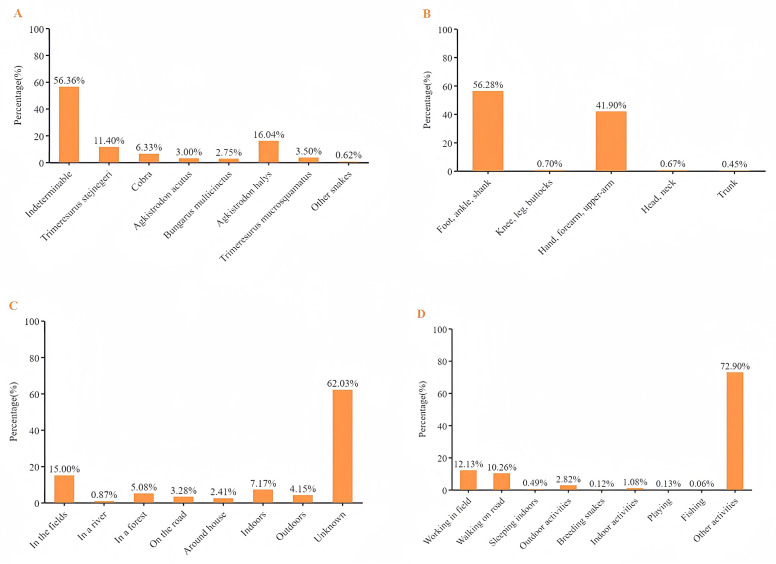
Snake species (A), site of bite (B), location (C), activity (D).

In this study, we retrospectively collected clinical data from 40,817 snakebite victims across 11 provinces in China from January 1, 2017, to December 31, 2021. The sociodemographic and epidemiological characteristics of snakebite victims are presented in Table 1. In our research, Jiangxi Province had the largest number of snakebite records (8861, 21.71%), followed by Hunan Province (5888, 14.43%) and Guizhou Province (4102, 10.05%). Over half (56.36%) of the snake species were indeterminate. Among the identified snake species, Agkistrodon halys (6547, 16.04%) and Trimeresurus stejnegeri (4655, 11.40%) were the most frequently reported (Fig 1). In addition, Agkistrodon halys was predominant in the Jiangxi and Hunan provinces, whereas Trimeresurus stejnegeri mainly occurred in the Guangdong, Hainan, and Fujian provinces (Table C in S1 File).

**Table 1:** The descriptive analysis of snakebite victims (*n* = 40,817).

Variables	*n* (%)	Variables	*n* (%)
**Outcome**		**Occupation**	
Healing	2785 (6.82)	Peasant	24368 (59.70)
Improvement	33651 (82.44)	Clerk	785 (1.92)
Unhealing	12 (0.03)	Student	648 (1.59)
Withdrawal	4346 (10.65)	Others	15016 (36.79)
Death	23 (0.06)		
**Sex**		**Marital status**	
Male	24637 (60.36)	Spinsterhood	4320 (10.58)
Female	16180 (39.64)	Married	35113 (86.03)
		Others	1384 (3.39)
			
**Age**		**Hospitalization duration**	
(0–6] years	735 (1.80)	< 3 days	16540 (40.52)
(6–17] years	1782 (4.36)	3-5 days	13679 (33.52)
(17–30] years	2338 (5.73)	> 5 days	10598 (25.96)
(30–40] years	3239 (7.94)		
(40–50] years	6717 (16.46)		
(50–65] years	16368 (40.10)		
> 65 years	9638 (23.61)		
			
**Province/Municipality/Autonomous Region**		**Snake specie**	
Hunan	5888 (14.43)	Indeterminable	23004 (56.36)
Hubei	1840 (4.51)	Trimeresurus stejnegeri	4655 (11.40)
Yunnan	2238 (5.48)	Naja atra	2583 (6.33)
Guizhou	4102 (10.05)	Deinagkistrodon acutus	1223 (3.00)
Guangdong	3881 (9.51)	Bungarus multicinctus	1123 (2.75)
Guangxi	3173 (7.77)	Agkistrodon halys	6547 (16.04)
Sichuan	2445 (5.99)	Protobothrops mangshanensis	1430 (3.50)
Jiangxi	8861 (21.71)	Other snakes	252 (0.62)
Chongqing	3454 (8.46)		
Hainan	2118 (5.19)		
Fujian	2817 (6.90)		
**Site of bite**		**Location**	
Foot, ankle, calf	22973 (56.28)	In farmlands	6124 (15.00)
Knee, thigh, buttock	284 (0.70)	In rivers	357 (0.87)
Hand, forearm, upper-arm	17102 (41.90)	In forests	2075 (5.08)
Head, neck	274 (0.67)	On roads	1339 (3.28)
Trunk	184 (0.45)	Around houses	984 (2.41)
		Indoors	2926 (7.17)
		Unrecorded	27012 (66.19)
**Activity**		**Envenomation on admission**	
Working in farmlands	4953 (12.13)	Mild	36007 (88.22)
Walking on roads	4189 (10.26)	Moderate	4550 (11.14)
Outdoor activities	1227 (3.01)	Severe	260 (0.64)
Indoor activities	643 (1.58)		
Breeding snakes	50 (0.12)		
Other activities	29755 (72.90)		
**Clinical manifestation**		**Medical treatment**	
Nephrotoxicity	32378 (79.32)	Antivenom treatments	28398 (69.57)
Neurotoxicity	6451 (15.80)	Supportive treatments	39648 (97.14)
Coagulopathy	19969 (48.92)	Traditional Chinese medicine treatments	33128 (81.16)
Inflammatory manifestations	6618 (16.21)	Local treatments	36443 (89.28)
Musculoskeletal dysfunction	11513 (28.21)		
**Prehospital delay time**			
[0–2] hours	13244 (32.45)		
(2, 4] hours	10190 (24.97)		
(4, 6] hours	4467 (10.94)		
(6, 12] hours	3689 (9.04)		
(12, 24] hours	4348 (10.65)		
> 24 hours	4879 (11.95)		

In this study, 60.36% of snakebite victims were males, 40.10% were aged 51–65, and 59.70% worked as peasants. The most common body parts bitten were the foot, ankle, and calf (56.28%), followed by the hand, forearm, and upper arm (41.90%). The exact locations of snakebite envenoming were not recorded for 66.19% of the snakebite victims, while among those recorded, the most common location was farmland (15.00%). Apart from other activities, snakebite envenoming were most frequent when working during farm work (12.13%) and walking on roads (10.26%), whereas snakebite envenoming owing to breeding snakes were rare (0.12%) (Fig 1). In addition, significant differences in snakebite exposure patterns were observed between adults and children (Table D in S1 File). Among the locations with clear records, snakebites in children were more likely to occur indoors (10.65%), whereas in adults, they were more commonly associated with farmlands (15.70%). Regarding activity types, snakebites in children were frequently linked to walking on roads (7.11%) and outdoor activities (5.56%), while in adults, they were more related to working in farmlands (12.66%) and walking on roads (10.47%).

**Fig 2:**
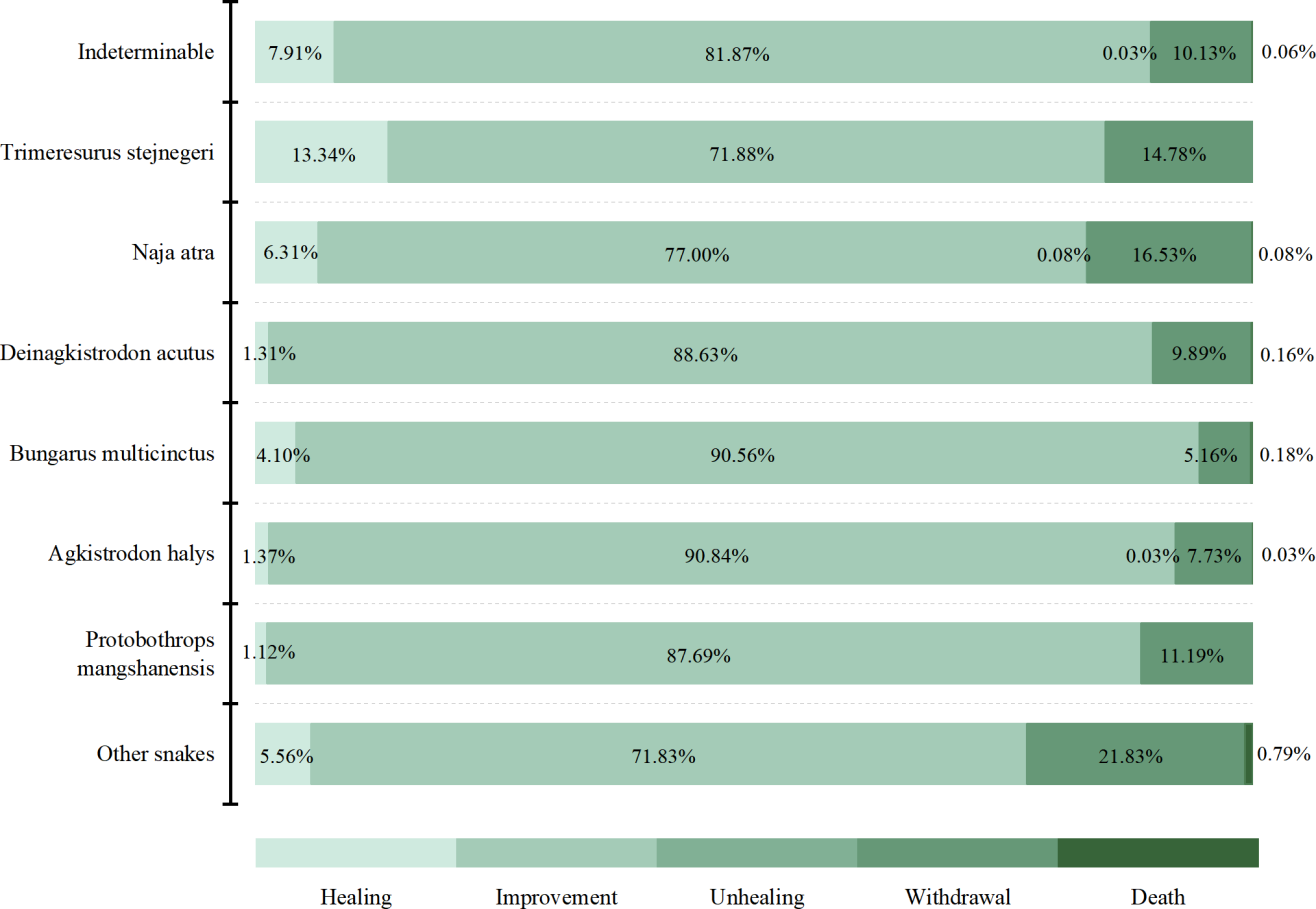
Association between snake species and discharge outcomes.

The discharge outcomes of snakebite victims in this study were categorized into five groups: healing (2785, 6.82%), improvement (33651, 82.44%), unhealing (12, 0.03%), withdrawal (4346, 10.65%), and death (23, 0.06%). Regarding the outcomes of victims bitten by Trimeresurus stejnegeri, wound healing or improvement accounted for 85.22% (3967), while giving up treatment accounted for 14.78% (688). Among those bitten by Agkistrodon halys, wound healing or improvement accounted for 92.21% (6037), while 7.73% (506) discontinued treatment (Fig 2).

**Fig 3:**
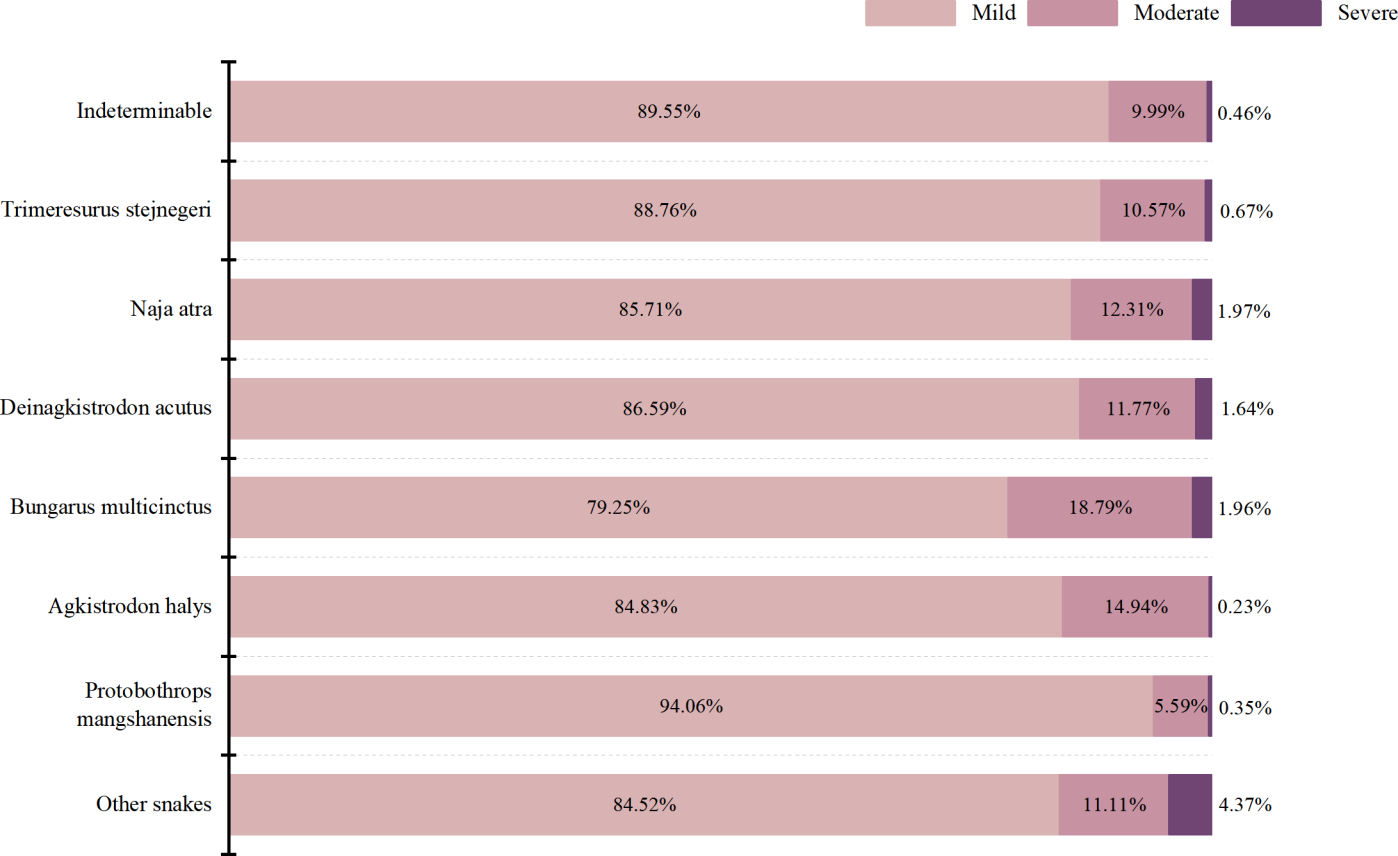
Association between snake species and degree of disease severity on admission.

We found that 88.22% of snakebite victims had mild envenomation on admission, 11.14% had moderate envenomation, and only 0.64% had severe envenomation. Furthermore, 88.76% of those bitten by Trimeresurus stejnegeri were mild envenomation, 10.57% and 0.67% were moderate envenomation and severe envenomation, respectively, upon admission. Among those bitten by Agkistrodon halys, the proportion of victims who experienced mild envenomation, moderate envenomation, and severe envenomation on admission were 84.83%, 14.94%, and 0.23%, respectively (Fig 3). Following snakebite envenoming, 79.32% of victims exhibited nephrotoxicity, about half of the victims coagulopathy, and only 15.80% showed neurotoxicity.

In this study, only 32.45% of snakebite victims sought hospitals care within 2 hours of the incident, while 22.60% experienced a prehospital delay exceeding 12 hours. Additionally, 40.52% of these victims were hospitalized for < 3 days, and 33.52% of remained hospitalized for 3–5 days. Most snakebite victims received supportive treatment (97.14%), local treatment (89.28%), Traditional Chinese Medicine treatment (81.16%). However, only antivenom treatment was recorded for 69.57% of the snakebite victims.

The results of the analysis indicated that snakebite victims with different discharge outcomes exhibited statistically significant differences in age, gender, occupation, marital status, hospitalization duration, snake specie, site of bite, location, activity, envenomation on admission, clinical manifestation, medical treatment, and prehospital delay time (*P* < 0.05) (Table E in S1 File). Factors significantly associated with discharge outcomes were included in the multivariate logistic regression analysis to identify the independent risk factors, with odds ratios (*OR*) and 95% confidence intervals (95% *CI*) (Table 2). The category with the smallest value was the reference for setting the dummy variable. Compared to victims aged 0–6 years, age ≥ 66 years old was a risk factor for discharge outcomes of snakebite victims (*OR* = 1.275, 95% *CI*: 1.020-1.593, *P* < 0.05). Discharge outcomes in clerks (*OR* = 1.391, 95% *CI*: 1.150-1.682, *P* < 0.005) and individuals in other professions (*OR* = 1.245, 95% *CI*: 1.176-1.318, *P* < 0.001) were typically more severe than peasants. Snakebite victims with 3–5 hospitalization duration (*OR* = 0.568, 95% *CI*: 0.534-0.605, *P* < 0.001) and over 5 days (*OR* = 0.322, 95% *CI*: 0.300-0.345, *P* < 0.001) demonstrated better discharge outcomes than those with less than 3 days. Additionally, snakebite envenoming in the trunk (*OR* = 0.491; 95% *CI*: 0.334-0.723, *P* < 0.001) were favorable for discharge outcomes compared to snakebites in the foot, ankle, and calf. Victims who experienced snakebite envenoming indoors (*OR* = 0.744, 95% *CI*: 0.648-0.855, *P* < 0.001) were more likely to improve than those bitten on farmlands. Compared with working on farmlands, victims bitten by snakes while walking on roads (*OR* = 0.695, 95% *CI*: 0.619-0.779, *P* < 0.001) and engaging in other activities (*OR* = 0.877, 95% *CI*: 0.791-0.972, *P* < 0.05) tended to have better discharge outcomes. Conversely, victims who were bitten during outdoor activities (*OR* = 1.489, 95% *CI*: 1.255-1.766, *P* < 0.001) and indoor activities (*OR* = 1.337, 95% *CI*: 1.043-1.714, *P* < 0.05) were more likely to experience deterioration. Snakebite victims with severe envenomation (*OR* = 2.777, 95% *CI*: 1.672-4.612, *P* < 0.001) were more likely to experience worse outcomes than those with mild envenomation. The presence of neurotoxicity (*OR* = 1.211, 95% *CI*: 1.126-1.303, *P* < 0.001) and coagulopathy (*OR* = 1.829, 95% *CI*: 1.728-1.936, *P* < 0.001) after snakebite envenoming were associated with a deterioration in discharge outcomes. Traditional Chinese Medicine treatments (*OR* = 0.754, 95% *CI*: 0.704-0.807, *P* < 0.001) and antivenom treatments (*OR* = 0.627, 95% *CI*: 0.591-0.665, *P* < 0.001) positively influenced the outcomes of snakebite victims. In contrast, victims who received supportive treatments (*OR* = 1.531, 95% *CI*: 1.306-1.796, *P* < 0.001) and local treatments (*OR* = 2.292, 95% *CI*: 2.103-2.499, *P* < 0.001) were more likely to experience negative discharge outcomes.

**Table 2:** The results of multivariate logistic regression analysis (*n* = 40,817).

Variables	*β*	*SE*	z	*P*	*OR*	95% *CI*	
Age							
(0–6] years	Ref						
(6–17] years	0.039	0.119	0.33	0.740	1.040	0.824	1.314
(17–30] years	-0.105	0.117	-0.90	0.366	0.900	0.716	1.131
(30–40] years	-0.051	0.119	-0.43	0.670	0.951	0.753	1.200
(40–50] years	0.094	0.115	0.82	0.415	1.098	0.877	1.376
(50–65] years	0.143	0.112	1.27	0.203	1.154	0.926	1.438
> 65 years	0.243	0.114	2.13	0.033	1.275	1.020	1.593
Sex							
Male	Ref						
Female	0.050	0.027	1.83	0.067	1.051	0.996	1.109
Occupation							
Peasant	Ref						
Clerk	0.330	0.097	3.40	0.001	1.391	1.150	1.682
Student	0.094	0.121	0.78	0.437	1.099	0.867	1.393
Others	0.219	0.029	7.53	< 0.001	1.245	1.176	1.318
Marital status							
Spinsterhood	Ref						
Married	-0.003	0.060	-0.05	0.958	0.997	0.886	1.121
Others	0.173	0.090	1.91	0.056	1.188	0.996	1.418
Hospitalization duration							
< 3 days	Ref						
3–5 days	-0.565	0.032	-17.56	< 0.001	0.568	0.534	0.605
> 5 days	-1.134	0.036	-31.61	< 0.001	0.322	0.300	0.345
Snake specie							
Indeterminable	Ref						
Trimeresurus stejnegeri	-0.198	0.044	-4.46	< 0.001	0.821	0.752	0.895
Naja atra	0.461	0.056	8.20	< 0.001	1.586	1.420	1.771
Deinagkistrodon acutus	0.491	0.077	6.36	< 0.001	1.634	1.404	1.900
Bungarus multicinctus	-0.370	0.085	-4.38	< 0.001	0.691	0.585	0.815
Agkistrodon halys	0.069	0.039	1.75	0.080	1.071	0.992	1.157
Protobothrops mangshanensis	0.509	0.070	7.24	< 0.001	1.663	1.449	1.908
Other snakes	0.648	0.157	4.13	< 0.001	1.911	1.405	2.599
Site of bite							
Foot, ankle, calf	Ref						
Knee, thigh, buttock	-0.049	0.159	-0.31	0.759	0.952	0.698	1.300
Hand, forearm, upper-arm	0.017	0.028	0.62	0.536	1.018	0.963	1.075
Head, neck	-0.299	0.161	-1.85	0.064	0.742	0.541	1.018
Trunk	-0.710	0.197	-3.61	< 0.001	0.491	0.334	0.723
Location							
In farmlands	Ref						
In rivers	0.132	0.140	0.94	0.345	1.142	0.867	1.503
In forests	-0.075	0.070	-1.07	0.285	0.928	0.809	1.064
On roads	0.052	0.084	0.62	0.534	1.054	0.894	1.242
Around houses	0.063	0.091	0.69	0.492	1.065	0.890	1.273
Indoors	-0.295	0.071	-4.18	< 0.001	0.744	0.648	0.855
Unrecorded	-0.441	0.050	-8.73	< 0.001	0.644	0.583	0.711
Activity							
Working in farmlands	Ref						
Walking on roads	-0.364	0.059	-6.21	< 0.001	0.695	0.619	0.779
Outdoor activities	0.398	0.087	4.57	< 0.001	1.489	1.255	1.766
Indoor activities	0.290	0.127	2.29	0.022	1.337	1.043	1.714
Breeding snakes	0.375	0.345	1.09	0.276	1.456	0.740	2.862
Other activities	-0.131	0.053	-2.50	0.013	0.877	0.791	0.972
Envenomation on admission							
Mild	Ref						
Moderate	0.380	0.197	1.93	0.053	1.462	0.995	2.150
Severe	1.021	0.259	3.95	< 0.001	2.777	1.672	4.612
Nephrotoxicity	-0.080	0.035	-2.31	0.021	0.923	0.862	0.988
Neurotoxicity	0.192	0.037	5.15	< 0.001	1.211	1.126	1.303
Coagulopathy	0.604	0.029	20.80	< 0.001	1.829	1.728	1.936
Inflammatory manifestations	-0.699	0.038	-18.48	< 0.001	0.497	0.461	0.535
Musculoskeletal dysfunction	-0.539	0.032	-17.09	< 0.001	0.583	0.548	0.620
Antivenom treatments	-0.467	0.030	-15.57	< 0.001	0.627	0.591	0.665
Supportive treatments	0.426	0.081	5.24	< 0.001	1.531	1.306	1.796
Traditional Chinese medicine treatments	-0.283	0.035	-8.17	< 0.001	0.754	0.704	0.807
Local treatments	0.830	0.044	18.88	< 0.001	2.292	2.103	2.499
Prehospital delay time	-0.000	0.000	-1.27	0.205	1.000	1.000	1.000

**Notes**: SE, Standard Error; OR, Odds Ratios; CI, Confidence Intervals.

## Discussion

In response to the global goal of halving the mortality and disability rates of snakebites by 2030, China has proactively sought to understand the epidemiological distribution, risks, and burdens associated with snakebite envenoming. To our knowledge, this study is the first multicenter, large-sample epidemiological survey on snakebite envenoming in China, and a total of 40817 snakebite victims were included in this study to explore the epidemiological characteristics of snakebite envenoming in China and the independent factors influencing hospital discharge outcomes among snakebite victims. Our research findings are expected to provide a reference for the future policies, interventions, and standard treatments of snakebite envenoming, thereby contributing to accomplish the strategic goal of halving the disability and mortality rates of snakebite envenoming by 2030 in China.

Among the 40817 snakebite victims, approximately 1/5 occurred in Jiangxi Province, 1/7 in Hunan Province, and 1/10 in Guizhou Province, constituting nearly half of the recorded cases. This distribution may be related to the geographical and climatic conditions. These provinces comprises over numerous mountains, hills, and rivers and possess a subtropical monsoon climate with a rich vegetation variety, providing excellent snake habitats [23–26]. Additionally, these three provinces have dense populations, high population density, and relatively underdeveloped economies primarily relying on agriculture. These factors contribute to increased snakebite incidents [9,28,29]. Notably, these three provinces are highly densely populated and relatively economically backward, with an economic structure still dominated by agriculture, leading to more snakebite events being recorded.

About snake species involved in the snakebite envenoming incident records, over half of the snakebite envenoming cases did not have information about the specific snakes responsible, posing challenges for subsequent wound management and administration of antivenom therapy. Agkistrodon halys and Trimeresurus stejnegeri accounted for the second-highest percentage, followed by unidentified snake species at 16.04% and 11.40%, respectively. This may be attributed to the Agkistrodon halys being China’s most widely distributed and numerous venomous snake species. In addition, Agkistrodon halys venom serves as a raw material for producing highly effective anticoagulant drugs, contributing to the high economic value of Agkistrodon halys cultivation. Alternatively, the Trimeresurus stejnegeri exhibits a strong arboreal nature, often hanging or coiling on tree branches. Owing to its vivid green coloration, the snake is not easily detected by people. As individuals traverse their habitats and inadvertently touch or approach these snakes, they frequently get bitten. Given the cold-resistant characteristics of the Agkistrodon halys family, the incubation cycle patterns of snakes, and the epidemiological distribution features observed in this study, we hypothesized that a significant proportion of unidentified snake species might belong to either the Agkistrodon halys or Trimeresurus stejnegeri categories. Considering their prevalence, hospitals should ensure an ample supply of antivenom for both Akistrodon halys and Trimeresurus stejnegeri. In cases where snake species cannot be identified immediately, using antivenom for Agkistrodon halys should be prioritized.

Regarding the demographic characteristics of snakebite victims, males outnumber females, and the most affected age group comprises those aged 50 years and older, primarily peasants. These findings are highly similar to epidemiological survey results from other regions [12,30,31]. According to traditional Chinese culture, the female gender occupies a less prominent place in manual work, since most women are limited to domestic tasks such as taking care of the house and the children, and therefore, they perform tasks with less risk of exposure to snakes [31]. Our study found that approximately 60% of the victims were bitten in the lower limbs, with over 50% of them being bitten below the knee, specifically on the feet, ankles, and calf, aligning with previous research findings [32,33]. The primary reason is likely accidental snakebite envenoming caused by agricultural or walking activities. Therefore, possible preventive measures include carrying a light source during outdoor activities, preferably wearing long-sleeved shirts and pants and high-top shoes, and avoiding skin exposure, especially when working in forests or fields during hot seasons [34,35]. When navigating through forests or dense vegetation, tools such as sticks can be used to probe the area before proceeding, ensuring no abnormalities [35].

There are significant differences in the snakebite exposure patterns between children and adults. Children’s daily activities are concentrated around their living areas (such as roads and indoors) and non-productive outdoor activities, making their risk of snakebites more associated with living scenarios. In contrast, adults’ exposure risks are closely related to their work environments due to occupational demands (such as farm work and forestry operations) and commuting (walking on roads). Therefore, it is necessary to enhance snake control measures around residential areas for children (e.g., clearing weeds, sealing gaps), while for adults, it is essential to strengthen occupational safety education and provide protective equipment.

It is widely recognized that snakebite envenoming inevitably results in envenomation. However, not every snakebite envenoming is accompanied by venom injection, resulting in severe envenomation. In this study, 88.22% of snakebite victims experienced mild envenomation, 11.14% had moderate envenomation, and only 0.64% exhibited severe envenomation on admission. Additionally, among the 40817 snakebite cases recorded in this study, over 80% of the victims were discharged with healing or improvement, whereas the mortality of snakebite victims was 0.06%. The severity of snakebite envenoming was significantly associated with the discharge outcomes of snakebite victims. As the severity increased, the discharge outcomes of the victims worsened.

Snake venom is a complex mixture of toxins and enzymes that induce distinct toxic effects, such as neurotoxicity, coagulopathy, and nephrotoxicity. Neurotoxicity include dizziness, blurred vision, ptosis, slurred speech, seizures, and etc. In severe cases, a significant blocking in neuromuscular conduction can cause respiratory muscle paralysis, ultimately leading to respiratory failure and death [36]. Following a snakebite envenoming, nephrotoxicity such as acute kidney injury and acute renal failure can also manifest. Insufficient renal perfusion, allergic reactions, and coagulation disorders can contribute to or exacerbate such clinical manifestation [37]. Additionally, coagulopathy are common in snakebite victims, ranging from mild bruising to severe coagulation dysfunction. This variation is owing to snake venom enzymes that can either promote or inhibit coagulation, leading to widespread bleeding or disseminated intravascular coagulation in severe cases [38].

Snakebite envenoming is medical emergency that typically require immediate medical intervention. The time between snakebite envenoming and the first medical care is an important variable that is directly related to high rates of therapeutic [31]. However, according to the findings of our study, prehospital delays were common. Only 32.45% of snakebite victims were able to go to hospitals to receive treatments within 2 hours post-bite, with an average prehospital delay time of 22.75 hours; the delay time exhibited significant variability. Some snakebite victims even experienced prehospital delays exceeding 100 hours. Severe prehospital delays predominantly stem from snakebite envenoming occurring in rural or remote mountainous areas with limited transportation, making it challenging for grassroots medical institutions or township health centers to accurately identify snakebite envenoming and provide appropriate treatments. Thereby, when snakebite victims reach comprehensive hospitals for standardized treatments, the optimal window for intervention is often missed. Additionally, another critical factor is the impact of the COVID-19 pandemic. Previous studies indicated that COVID-19 exacerbated the economic burden in areas with high snakebite burdens. Concurrently, the pandemic altered healthcare-seeking behaviors, as numerous individuals avoided formal medical facilities during outbreaks due to infection fears, instead opting for traditional practitioners [39–41].

Nevertheless, the subsequent treatment outcomes for snakebite victims in this study were gratifying, with 97.14% of the snakebite victims receiving supportive treatments, 89.28% undergoing local treatments, and 81.16% receiving Traditional Chinese Medicine treatments. Previous research indicates that the efficacy of Traditional Chinese Medicine primarily lies in its antivenom, analgesic, anti-inflammatory, and antibacterial properties, which can effectively prevent damage to vital organs and achieve desirable clinical treatment results [42]. Future intervention measures should focus on geographic regions or clusters with high susceptibility and limited healthcare resources. It is crucial to enhance public awareness of snakebite envenoming prevention and first aid in these areas, aiming to minimize the occurrence of snakebite envenoming incidents and reduce prehospital delays [43]. Furthermore, enhancing the ability of medical staff to correctly identify and manage snakebite envenoming can significantly reduce disability rates, mortality, and other adverse outcomes in snakebite victims [44].

Antivenom is the sole specific therapeutic approach, and its mechanism of action involves neutralizing snake venom by binding to antigen-antibody reactions [45]. In this study, only 69.57% of snakebite victims received antivenom treatments. Despite rising demand, the production cycle of antivenom is lengthy, and profits are low, leading to an insufficient increase in output [46,47]. Additionally, the stringent storage requirement of maintaining the serum between 2–8°C poses challenges, particularly for resource-limited regions, in ensuring timely availability for snakebite emergencies [48]. However, snakebite envenoming often occur in economically and medically underdeveloped areas, and are relatively scattered, making it impractical to ensure that every primary or community hospital stores an antivenom. Despite challenges, it remains essential to ensure that snakebite victims receive prompt and effective treatment within 1–2 hours in the future. Furthermore, the occurrence of snakebite envenoming both temporally and geographically dispersed. Therefore, this study aimed to provide a reference for the supply and distribution of antivenom through a multicenter, large-sample survey.

The designation of the snakebite envenoming as an NTD by the WHO is highly appropriate. Our survey across 11 provinces with tropical or subtropical monsoon climates revealed a less-than-optimistic scenario regarding the snakebite envenoming in China. Therefore, policymakers in China, particularly those in less developed provinces, should fully consider the burden of snakebite envenoming, which includes high disability, low mortality, serious underreporting, gross disparities in distribution, and high cost-effectiveness in control. Additionally, more research on snakebite envenoming is necessary, particularly the establishment of a comprehensive database, which will ensure a more accurate assessment of the burden of snakebite envenoming in China [11].

Several factors influence the discharge outcomes of snakebite victims. Compared to snakebite victims aged 0–6 years, those aged > 66 years had poorer discharge outcomes, likely because of the increased likelihood of various chronic diseases with advancing age. Surprisingly, our study found that snakebite victims in occupations such as clerks or other professions faced a higher risk of poorer discharge outcomes than peasants. We hypothesize that this could stem from their limited knowledge of snake species and emergency procedures. Consequently, they might not promptly extract or suction the venom and reduce their activity when bitten, potentially leading to more severe consequences. Future research in other regions could further investigate individual snakebite envenoming knowledge levels. Regarding where the snakebite envenoming occurred, those bitten indoors may have better outcomes than those bitten on farmlands, as most venomous snakes prefer habitats such as fields and forests [7]. Furthermore, Traditional Chinese Medicine and antivenom treatments have demonstrated a significantly positive impact on the outcomes of snakebite victims, while supportive and local treatments yield the opposite effects. This disparity may be attributed to the severity of the condition in snakebite victims receiving supportive treatment. In contrast, those undergoing local treatments may have larger or poorly healing wounds, potentially leading to recovery failure.

This study reflects several critical deficiencies in the healthcare system’s capacity for snakebite management, particularly the uneven distribution of primary healthcare resources in rural endemic regions such as Jiangxi, Hunan, and Guizhou provinces, where shortages of antivenom stocks and inadequate emergency response capabilities remain prevalent. Furthermore, the substantial supply chain limitations are evidenced by the finding that only 69.57% of snakebite victims received antivenom therapy, reflecting systemic challenges in production, distribution, and equitable allocation of this essential treatment. These interconnected issues collectively undermine effective snakebite management and highlight the need for comprehensive health system strengthening. Therefore, this study recommends incorporating snakebite management into the national basic public health service package, mandating antivenom stockpiling in primary hospitals in high-incidence areas, or establishing regional antivenom storage centers. Lessons could also be learned from foreign experiences in increasing antivenom production capacity through public–private partnership (PPP) models [49]. For example, in India, the PPP model has been successfully applied in various healthcare sectors, including providing medical services for impoverished populations. Through this model, the government can collaborate with the private sector to enhance antivenom production capacity and supply efficiency. Such collaboration not only addresses the shortage of antivenom but also improves the overall quality and accessibility of medical services through public–private cooperation. Additionally, calls for multi-departmental collaboration to establish a Chinese Expert Consultation Committee on Snakebite Envenoming, set up provincial research centers in high-incidence provinces, and organize regional rescue and research networks by these centers. Regular training and public education programs should be organized, and relevant scientific research should be conducted. Dedicated snakebite envenoming sessions should also be held at major conferences to enhance the awareness of emergency medical personnel regarding snakebite treatment.

We acknowledge some limitations in generalizing the findings of our study. First, this study is a cross-sectional retrospective study, and the reported cases of 40817 snakebite victims were collected through a multistage stratified cluster random sampling in 11 provinces of China. These cases represented only recorded instances of snakebite envenoming and snakebite victims resulting in death or disability within the selected sampling units. Second, we cannot ignore the impact of the COVID-19 pandemic, which lasted almost 3 years. The impact of the COVID-19 pandemic on the healthcare system in China, especially in outpatient and emergency departments, most likely to receive snakebite victims, and the strict “unsilent” system of management in China may have influenced the failure to enroll a portion of snakebite victims in this study. Third, the lack of uniformity in medical history recording formats across different hospitals limits the detail description of symptoms and signs. Combined with methodological constraints, these result in an absence of detailed classification and description of clinical manifestations, thereby categorizing various clinical manifestations into a single systemic effect in the analysis. This limitation highlights the need for more precise case definitions and classification systems in future research to enhance the accuracy and reliability of epidemiological and clinical analyses. Additionally, a limitation of this study is its reliance on hospital-based data collection, resulting in an inability to fully reflect the broader population. Various community-level factors have not been adequately considered. However, these factors are crucial for a comprehensive understanding of health outcomes. Therefore, future research should integrate hospital data with community survey data to provide a more comprehensive and accurate analysis.

## Conclusions

This study was the first multicenter, large-sample survey using a multistage stratified cluster random sampling to assess the prevalence of snakebite envenoming in China. It provides epidemiologic insights into 40817 snakebite victims across 11 provinces in China. This research addresses a notable gap in the epidemiological study of snakebite envenoming in China and the study findings are expected to provide a reference for the future policies, interventions, and standard treatments of snakebite envenoming, thereby contributing to accomplish the strategic goal of halving the disability and mortality rates of snakebite envenoming by 2030 in China. Several targeted management and prevention measures could mitigate snakebite envenoming in China, such as improving infrastructure and resources in health facilities, developing guidelines for the treatment of snakebite envenoming, strengthening the cultivation of all health workers, and providing a sustainable supply of safe, effective, and affordable antivenom, thereby ensuring that snakebite victims have access to fair and accurate diagnosis and treatment.

## Supporting information

S1 File Including: Methods-detailed definitions of major variables of **Table B.** Snakebite envenoming Datasheet. **Table A.** The number of hospitals in each province. **Table B.** Snakebite envenoming Datasheet. **Table C.** The regional distribution characteristics of snakes (*n* = 40,817). **Table D.** Differences in snakebite exposure pattern between adults and children. **Table E.** The results of the significant difference analysis (*n* = 40,817).
(DOCX)
