# Supplementary material for: Clinical epidemiology of snakebite envenoming in hospitals 11 provinces of Yangtze River Basin and southern regions of China: A retrospective hospital-based analysis
Source: PLoS Negl Trop Dis. 2026 Apr 27;20(4):e0013247. doi: 10.1371/journal.pntd.0013247 (PMC13119834; doi:10.1371/journal.pntd.0013247)
Supplement: S1 File — Including: Methods-detailed definitions of major variables of Table B. Snakebite envenoming Datasheet. Table A. The number of hospitals in each province. Table B. Snakebite envenoming Datasheet. Table C. The regional distribution characteristics of snakes (n = 40,817). Table D. Differences in snakebite exposure pattern between adults and children. Table E. The results of the significant difference analysis (n = 40,817). (DOCX) [file pntd.0013247.s001.docx]

**Supporting Information**

**1. Methods-detailed definitions of major variables of Table B. Snakebite envenoming Datasheet**

**Outcome**: snakebite victims are considered **Healing** when the lesion is resolved, symptoms have disappeared, and abnormal findings from relevant examinations and laboratory tests have returned to normal. **Improvement** is defined as a reduction or containment of the lesion, alleviation of symptoms, and amelioration of abnormal test results. All other outcomes are classified as **Unhealing**. If a snakebite victim discharges themselves prematurely and does not continue with the treatment regimen, this is regarded as **Withdrawal** [1].

**Envenomation on admission**: The envenoming severity on admission was assessed according to the Simplified Clinical Severity Assessment Scale for Snakebites. The classification is as follows: (1) **Mild envenoming**, presenting dry bite or only local symptoms around the wound, such as pain, bleeding from the wound, skin bruising, non-progressive limb swelling not exceeding the elbow or knee, and no progress in 12 h after the bite; local sensory abnormalities, numbness, neuropathic pain, muscle spasm, and fasciculation; (2) **Moderate envenoming**, characterized by progressive swelling, purpura, or ecchymosis away from the wound, not exceeding half of the limb; non-fatal systemic symptoms or signs, such as nausea, vomiting, hematemesis/melena; mild perioral numbness, mild fatigue, respiratory muscle weakness resulting in dyspnea or other respiratory discomfort, language barriers, diplopia, hearing or taste abnormalities, and difficulty swallowing; slight changes in laboratory results (<2 times the upper limit of normal), mild abnormalities in coagulation function but no systemic bleeding; (3) **Severe envenoming**, manifesting local symptoms such as widespread swelling, erythema, or ecchymosis rapidly progressing beyond more than half of the body, or bites on the head, neck, or trunk; obvious symptoms and signs such as fatigue or progressive muscle weakness, blurred vision, ptosis, altered consciousness, tachycardia, shortness of breath or distress, or the need for mechanical ventilation support, hemodynamic instability (such as hypotension or shock), suspected or confi rmed intracranial or abdominal bleeding; laboratory results showing signifi cant abnormalities, such as severe coagulopathy with bleeding in areas other than the wound, thrombocytopenia, signifi cant abnormalities in muscle enzymes (≥2 times the upper limit of normal) [2].

**Clinical manifestations** [1, 3]:

**Nephrotoxicity**: The presence of any one of the following observable or palpable indicators of disease or bodily condition is considered to indicate nephrotoxicity in the snakebite victim: Swelling, Edema.

**Neurotoxicity**: The presence of any one of the following observable or palpable indicators of disease or bodily condition is considered to indicate neurotoxicity in the snakebite victim: Numbness, Mild hypesthesia, Hypesthesia, Muscle weakness, Altered mental status, Acute facies, Distressed facies.

**Coagulopathy**: The presence of any one of the following observable or palpable indicators of disease or bodily condition is considered to indicate coagulopathy in the snakebite victim: Bleeding, Oozing, Minor blood staining, Blood crust, Scab, Bruise, Cyanosis, Subcutaneous hemorrhage, Bite mark, Laceration, Pallor, Pale, Ashen pallor.

**Inflammatory manifestations**: The presence of any one of the following observable or palpable indicators of disease or bodily condition is considered to indicate inflammatory manifestations in the snakebite victim: Necrosis, Ischemia, Black discoloration, Jet-black, Dark brown, Dusky red, Purulent discharge, Yellow discharge, Yellowish exudate , Pus drainage, Ulceration, Skin breakdown, Erythema with warmth, Atrophy, Induration, Vesicle, Exudate.

**Musculoskeletal dysfunction**: The presence of any one of the following observable or palpable indicators of disease or bodily condition is considered to indicate musculoskeletal dysfunction in the snakebite victim: Tenderness, Pain on palpation, Cold skin, Increased skin turgor, Localized hypertonia, Hypokinesia, Restricted mobility, Cruciate incision.

**Medical treatment** [1, 3]:

**Supportive treatments**: The following treatment measures are included: Symptomatic and supportive care, Electrolyte balance maintenance, Continuous ECG monitoring, Hepatorenal protection, Myocardial metabolic support, Anti-infective therapy, Tetanus prophylaxis, Anti-inflammatory therapy, Corticosteroid therapy, Antiallergic therapy, Hemodialysis, Blood purification, Endotracheal intubation, Analgesia, Gastroprotection.

**Traditional Chinese medicine treatments**: The following treatment measures are included: Diuretic detoxification therapy, Heat-clearing and detoxifying therapy, Cooling blood and resolving stasis Hemostasis-promoting and blood-activating therapy, Jidesheng anti-venom tablets, Blood-activating and stasis-resolving therapy, Blood-vitalizing and swelling-reducing treatment.

**Antivenom treatments**: Antivenom.

**Local treatments**: The following treatment measures are included: Local block therapy, Negative pressure wound therapy, Incisional decompression, Debridement, Limb elevation, Improve microcirculation, Magnesium sulfate wet compress, Decongestion.

**References:**

1. Lai RD. Diagnosis and treatment of snakebite. Beijing: Scientific and Technical documentation press; 2023.

2. Lai RD, Yan SJ, Wang SJ, Yang SQ, Yan ZR, Lan P, et al. The Chinese guideline for management of snakebites. World Journal of Emergency Medicine. 2024;15(5):333-55. doi: 10.5847/wjem.j.1920-8642.2024.076. PubMed PMID: WOS:001340432500011.

3. David A Warrell. Guidelines for the management of snake-bites. 1st ed. World Health Organization: Regional Office for South-East Asia; 2010.

**2Table A.** **The number of hospitals in each province**

| **Province** | **Hospital (*n*)** | |
| --- | --- | --- |
|  | **Tertiary Grade A** | **Tertiary Grade B** |
| Hunan | 5 | 6 |
| Hubei | 8 | 4 |
| Yunnan | 8 | 6 |
| Guizhou | 6 | 8 |
| Guangdong | 5 | 3 |
| Guangxi | 4 | 3 |
| Sichuan | 7 | 4 |
| Jiangxi | 5 | 9 |
| Chongqing | 7 | 3 |
| Hainan | 3 | 5 |
| Fujian | 3 | 4 |
| **Total** | 61 | 55 |

**Table B. Snakebite envenoming Datasheet**

| Variables | |  |
| --- | --- | --- |
| Demographic and Sociological Characteristics | patient’s identification number |  |
|  | department |  |
|  | province |  |
|  | sex |  |
|  | age |  |
|  | occupation |  |
|  | marital status |  |
|  | contact person |  |
|  | contact Person’s phone number |  |
| Past Medical History | |  |
| Discharge Diagnosis | |  |
| Outcome | |  |
| Hospitalization Duration | |  |
| Characteristics of Snakebite Envenoming | snake specie |  |
|  | site of bite |  |
|  | location |  |
|  | activity |  |
|  | envenomation on admission |  |
| Clinical Manifestation | |  |
| Medical Treatment | |  |
| Dose of Antivenom Used | |  |
| Type of Antivenom | |  |
| Prehospital Delay Time | |  |

**Table C. The regional distribution characteristics of snakes (*n* = 40,817)**

| **Snake**  **Province** | **Hunan** | **Hubei** | **Yunnan** | **Guizhou** | **Guangdong** | **Guangxi** | **Sichuan** | **Jiangxi** | **Chongqing** | **Hainan** | **Fujian** |
| --- | --- | --- | --- | --- | --- | --- | --- | --- | --- | --- | --- |
| Indeterminable | 2533  (11.00%) | 1714  (7.45%) | 1757  (7.64%) | 3465  (15.06%) | 1862  (8.09%) | 1357  (5.90%) | 2280  (9.91%) | 2370  (10.30%) | 3176  (13.81%) | 946  (4.11%) | 1544  (6.71%) |
| Trimeresurus stejnegeri | 101  (2.17%) | 10  (0.21%) | 327  (7.02%) | 283  (6.08%) | 1018  (21.87%) | 526  (11.30%) | 23  (0.49%) | 568  (12.20%) | 16  (0.34%) | 908  (19.51%) | 875  (18.80%) |
| Naja atra | 522  (20.21%) | 7  (0.27%) | 68  (2.63%) | 24  (0.93%) | 514  (19.90%) | 643  (24.89%) | 15  (0.58%) | 492  (19.05%) | 6  (0.23%) | 205  (7.94%) | 87  (3.37%) |
| Deinagkistrodon acutus | 294  (24.04%) | 24  (1.96%) | 2  (0.16%) | 89  (7.28%) | 14  (1.14%) | 260  (21.26%) | 0  (0.00%) | 375  (30.66%) | 10  (0.82%) | 1  (0.08%) | 154  (12.59%) |
| Bungarus multicinctus | 111  (9.88%) | 5  (0.45%) | 13  (1.16%) | 3  (0.27%) | 89  (7.93%) | 153  (13.62%) | 1  (0.09%) | 703  (62.60%) | 2  (0.18%) | 29  (2.58%) | 14  (1.25%) |
| Agkistrodon halys | 2085  (31.85%) | 49  (0.75%) | 67  (1.02%) | 156  (2.38%) | 36  (0.55%) | 60  (0.92%) | 40  (0.61%) | 3992  (60.97%) | 43  (0.66%) | 14  (0.21%) | 5  (0.08%) |
| Protobothrops mangshanensis | 225  (15.73%) | 30  (2.10%) | 2  (0.14%) | 66  (4.62%) | 214  (14.97%) | 139  (9.72%) | 81  (5.66%) | 353  (24.69%) | 192  (13.43%) | 0  (0.00%) | 128  (8.95%) |
| Other snakes | 17  (6.75%) | 1  (0.40%) | 2  (0.79%) | 16  (6.35%) | 134  (53.17%) | 35  (13.89%) | 5  (1.98%) | 8  (3.17%) | 9  (3.57%) | 15  (5.95%) | 10  (3.97%) |

**Table D. Differences in snakebite exposure pattern between adults and children**

| **Variables** | **Children (0-17 years)**  **(*n* = 2517)** | **Adults (**> **17 years)**  **(*n* = 38300)** | ***χ*^2^** | ***P*** |
| --- | --- | --- | --- | --- |
| Location (N, %) |  |  |  |  |
| In farmlands | 110 (4.37) | 6014 (15.70) | 405.123 | < 0.001 |
| In rivers | 26 (1.03) | 331 (0.86) |  |  |
| In forests | 107 (4.25) | 1968 (5.14) |  |  |
| On roads | 172 (6.82) | 1167 (3.05) |  |  |
| Around houses | 113 (4.49) | 871 (2.27) |  |  |
| Indoors | 268 (10.65) | 2658 (6.94) |  |  |
| Unrecorded | 1721 (68.38) | 25291 (66.03) |  |  |
| Activity (N, %) |  |  |  |  |
| Working in farmlands | 105 (4.17) | 4848 (12.66) | 259.205 | < 0.001 |
| Walking on roads | 179 (7.11) | 4010 (10.47) |  |  |
| Outdoor activities | 140 (5.56) | 1087 (2.84) |  |  |
| Indoor activities | 29 (1.15) | 614 (1.60) |  |  |
| Breeding snakes | 1 (0.04) | 49 (0.13) |  |  |
| Other activities | 2063 (81.96) | 27692 (72.30) |  |  |

**Table E. The results of the significant difference analysis (*n* = 40,817)**

| Variables | Outcome | | | | | *χ*^2^*/H* | *P* |
| --- | --- | --- | --- | --- | --- | --- | --- |
|  | Healing | Improvement | Unhealing | Withdrawal | Death |  |  |
| Age (N, %) | | | | | | | |
| (0-6] years | 61 (2.19) | 590 (1.75) | 1 (8.33) | 83 (1.91) | 0 (0.00) | 234.554 | < 0.001 |
| (6-17] years | 135 (4.85) | 1459 (4.34) | 1 (8.33) | 186 (4.28) | 1 (4.35) |  |  |
| (17-30] years | 245 (8.80) | 1835 (5.45) | 0 (0.00) | 255 (5.87) | 3 (13.04) |  |  |
| (30-40] years | 306 (10.99) | 2590 (7.70) | 1 (8.33) | 340 (7.82) | 2 (8.70) |  |  |
| (40-50] years | 54 (18.46) | 5449 (16.19) | 0 (0.00) | 753 (17.33) | 1 (4.35) |  |  |
| (50-65] years | 1119 (40.17) | 13463 (40.01) | 6 (50.01) | 1775 (40.84) | 5 (21.74) |  |  |
| > 65 years | 405 (14.54) | 8265 (24.56) | 3 (25.00) | 954 (21.95) | 11 (47.82) |  |  |
| Sex (N, %) | | | | | | | |
| Male | 1663 (59.71) | 20406 (60.64) | 9 (75.00) | 2543 (58.51) | 16 (69.57) | 9.673 | 0.046 |
| Female | 1122 ( 40.29) | 13245 (39.36) | 3 (25.00) | 1803 (41.49) | 7 (30.43) |  |  |
| Occupation (N, %) | | | | | | | |
| Peasant | 1832 (65.78) | 20038 (59.55) | 8 (66.67) | 2477 (56.99) | 13 (56.52) | 83.525 | < 0.001 |
| Clerk | 28 (1.01) | 691 (2.05) | 0 (0.00) | 66 (1.52) | 0 (0.00) |  |  |
| Student | 54 (1.94) | 521 (1.55) | 1 (8.33) | 72 (1.66) | 0 (0.00) |  |  |
| Others | 871 (31.27) | 12401 (36.85) | 3 (25.00) | 1731 (39.83) | 10 (43.48) |  |  |
| Marital status (N, %) | | | | | | | |
| Spinsterhood | 322 (11.56) | 3571 (10.61) | 3 (25.00) | 422 (9.71) | 0 (0.00) |  |  |
| Married | 2419 (86.86) | 28889 (85.85) | 9 (75.00) | 3779 (86.95) | 17 (73.91) | 51.318 | < 0.001 |
| Other | 44 (1.58) | 1191 (3.54) | 0 (0.00) | 145 (3.34) | 4 (17.39) |  |  |
| Hospitalization duration (N, %) | | | | | | | |
| <3 days | 1110 (39.86) | 12467 (37.05) | 8 (66.66) | 2942 (67.69) | 13 (56.53) | 1916.232 | < 0.001 |
| 3-5 days | 551 (19.78) | 12241 (36.37) | 2 (16.67) | 878 (20.20) | 7 (30.43) |  |  |
| >5 days | 1124 (40.36) | 8943 (26.58) | 2 (16.67) | 526 (12.11) | 3 (13.04) |  |  |
| Snake specie (N, %) | | | | | | | |
| Indeterminable | 1819 (65.31) | 18833 (55.97) | 8 (66.66) | 2331 (53.64) | 13 (56.50) | 1220.845 | < 0.001 |
| Trimeresurus stejnegeri | 621 (22.31) | 3346 (9.94) | 0 (0.00) | 688 (15.83) | 0 (0.00) |  |  |
| Naja atra | 163 (5.85) | 1989 (5.91) | 2 (16.67) | 427 (9.83) | 2 (8.70) |  |  |
| Deinagkistrodon acutus | 16 (0.57) | 1084 (3.22) | 0 (0.00) | 121 (2.78) | 2 (8.70) |  |  |
| Bungarus multicinctus | 46 (1.65) | 1017 (3.02) | 0 (0.00) | 58 (1.33) | 2 (8.70) |  |  |
| Agkistrodon halys | 90 (3.24) | 5947 (17.67) | 2 (16.67) | 506 (11.64) | 2 (8.70) |  |  |
| Protobothrops mangshanensis | 16 (0.57) | 1254 (3.73) | 0 (0.00) | 160 (3.68) | 0 (0.00) |  |  |
| Other snakes | 14 (0.50) | 181 (0.54) | 0 (0.00) | 55 (1.27) | 2 (8.70) |  |  |
| Site of bite (N, %) | | | | | | | |
| Foot, ankle, calf | 1416 (50.84) | 19341 (57.48) | 5 (41.67) | 2198 (50.58) | 13 (56.52) | 168.471 | < 0.001 |
| Knee, thigh, buttock | 22 (0.79) | 228 (0.68) | 0 (0.00) | 34 (0.78) | 0 (0.00) |  |  |
| Hand, forearm, upper-arm | 1277 (45.86) | 13744 (40.84) | 7 (58.33) | 2065 (47.51) | 9 (39.13) |  |  |
| Head, neck | 36 (1.29) | 206 (0.61) | 0 (0.00) | 32 (0.74) | 0 (0.00) |  |  |
| Trunk | 34 (1.22) | 132 (0.39) | 0 (0.00) | 17 (0.39) | 1 (4.35) |  |  |
| Location (N, %) | | | | | | | |
| In farmlands | 240 (8.62) | 5090 (15.13) | 2 (16.67) | 788 (18.13) | 4 (17.39) | 431.090 | < 0.001 |
| In rivers | 13 (0.47) | 287 (0.85) | 0 (0.00) | 57 (1.31) | 0 (0.00) |  |  |
| In forests | 211 (7.58) | 1493 (4.44) | 0 (0.00) | 370 (8.51) | 1 (4.35) |  |  |
| On roads | 48 (1.72) | 1144 (3.40) | 0 (0.00) | 146 (3.36) | 1 (4.35) |  |  |
| Around houses | 59 (2.12) | 757 (2.25) | 0 (0.00) | 168 (3.87) | 0 (0.00) |  |  |
| Indoors | 158 (5.67) | 2502 (7.43) | 0 (0.00) | 264 (6.07) | 2 (8.70) |  |  |
| Unrecorded | 2056 (73.82) | 22378 (66.50) | 10 (83.33) | 2553 (58.75) | 15 (65.21) |  |  |
| Activity (N, %) | | | | | | | |
| Working in farmlands | 239 (8.58) | 3983 (11.84) | 0 (0.00) | 726 (16.71) | 5 (21.74) | 746.561 | < 0.001 |
| Walking on roads | 570 (20.47) | 3050 (9.06) | 2 (16.67) | 564 (12.97) | 3 (13.04) |  |  |
| Outdoor activities | 106 (3.81) | 870 (2.59) | 0 (0.00) | 251 (5.78) | 0 (0.00) |  |  |
| Indoor activities | 35 (1.26) | 507 (1.51) | 0 (0.00) | 100 (2.30) | 1 (4.35) |  |  |
| Breeding snakes | 3 (0.11) | 35 (0.10) | 0 (0.00) | 12 (0.28) | 0 (0.00) |  |  |
| Other activities | 1832 (65.77) | 25206 (74.90) | 10 (83.33) | 2693 (61.96) | 14 (60.87) |  |  |
| Envenomation on admission (N, %) | | | | | | | |
| Mild | 2306 (82.80) | 29823 (88.63) | 8 (66.67) | 3857 (88.75) | 13 (56.52) | 649.694 | < 0.001 |
| Moderate | 447 (16.05) | 3649 (10.84) | 3 (25.00) | 450 (10.35) | 1 (4.35) |  |  |
| Severe | 32 (1.15) | 179 (0.53) | 1 (8.33) | 39 (0.90) | 9 (39.13) |  |  |
| Nephrotoxicity (N, %) | | | | | | | |
| No | 706 (25.35) | 6628 (19.70) | 5 (41.67) | 1091 (25.10) | 9 (39.13) | 116.739 | < 0.001 |
| Yes | 2079 (74.65) | 27023 (80.30) | 7 (58.33) | 3255 (74.90) | 14 (60.97) |  |  |
| Neurotoxicity (N, %) | | | | | | | |
| No | 2653 (95.26) | 27822 (82.68) | 8 (66.67) | 3866 (88.96) | 17 (73.91) | 393.058 | < 0.001 |
| Yes | 132 (4.74) | 5829 (17.32) | 4 (33.33) | 480 (11.04) | 6 (26.09) |  |  |
| Coagulopathy (N, %) | | | | | | | |
| No | 2042 (73.32) | 16657 (49.50) | 6 (50.00) | 2129 (48.99) | 14 (60.87) | 593.479 | < 0.001 |
| Yes | 743 (26.68) | 16994 (50.50) | 6 (50.00) | 2217 (51.01) | 9 (39.13) |  |  |
| Inflammatory manifestations (N, %) | | | | | | | |
| No | 1998 (71.74) | 29223 (83.87) | 9 (75.00) | 3949 (90.87) | 20 (86.96) | 458.753 | < 0.001 |
| Yes | 787 (28.26) | 5428 (16.13) | 3 (25.00) | 397 (9.13) | 3 (13.04) |  |  |
| Musculoskeletal dysfunction (N, %) | | | | | | | |
| No | 2094 (75.19) | 23279 (69.18) | 8 (66.67) | 3906 (89.88) | 17 (73.91) | 831.475 | < 0.001 |
| Yes | 691 (24.81) | 10372 (30.82) | 4 (33.33) | 440 (10.12) | 6 (26.09) |  |  |
| Antivenom treatments (N, %) | | | | | | | |
| No | 769 (27.61) | 9960 (29.60) | 3 (25.00) | 1676 (38.56) | 11 (47.83) | 160.747 | < 0.001 |
| Yes | 2016 (72.39) | 23691 (70.40) | 9 (75.00) | 2670 (61.44) | 12 (52.17) |  |  |
| Supportive treatments (N, %) | | | | | | | |
| No | 61 (2.19) | 1092 (3.25) | 1 (8.33) | 13 (0.30) | 2 (8.70) | 128.982 | < 0.001 |
| Yes | 2724 (97.81) | 32559 (96.75) | 11 (91.67) | 4333 (99.70) | 21 (91.30) |  |  |
| Traditional Chinese medicine treatments (N, %) | | | | | | | |
| No | 672 (24.13) | 5657 (16.81) | 3 (25.00) | 1351 (31.09) | 6 (26.09) | 568.962 | < 0.001 |
| Yes | 2113 (75.87) | 27994 (83.19) | 9 (75.00) | 2995 (68.91) | 17 (73.91) |  |  |
| Local treatments (N, %) | | | | | | | |
| No | 885 (31.78) | 3017 (8.97) | 2 (16.67) | 464 (10.68) | 6 (26.09) | 1405.081 | < 0.001 |
| Yes | 1900 (68.22) | 30634 (91.03) | 10 (83.33) | 3882 (89.32) | 17 (73.91) |  |  |
| Prehospital delay time [M（P_25_, P_75_)] | 4.30 (2.21,11.93) | 3.00 (2.00,10.00) | 15.25 (2.25,30.57) | 3.17 (2.00,9.00) | 12.15 (5.00,24.00) | 140.480 | < 0.001 |
